# The contribution of non-physician clinicians to the provision of surgery in rural Zambia—a randomised controlled trial

**DOI:** 10.1186/s12960-019-0398-9

**Published:** 2019-07-22

**Authors:** Jakub Gajewski, Mweene Cheelo, Leon Bijlmakers, John Kachimba, Chiara Pittalis, Ruairi Brugha

**Affiliations:** 1 Institute of Global Surgery, Royal College of Surgeons in Ireland, 123 St Stephens Green, Dublin 2, Ireland; 2 Department of Surgery, Surgical Society of Zambia, University Teaching Hospital, P.O. Box, 50110 Lusaka, Zambia; 3 Radboud University Medical Centre Netherlands, Geert Grooteplein Zuid 10, 6525 GA Nijmegen, Netherlands; 4 Division of Population Health Sciences, Royal College of Surgeons in Ireland, 123 St Stephens Green, Dublin 2, Ireland

**Keywords:** Surgery, Hernia repair, Clinical officers, Malawi, Global surgery, Task-shifting, Non-physician clinicians, Zambia, Medical licentiates, Patient outcomes

## Abstract

**Background:**

The global shortage of surgeons disproportionately impacts low- and middle-income countries. To mitigate this, Zambia introduced a ‘task-shifting’ solution and started to train non-physician clinicians (NPCs) called medical licentiates (ML) to perform surgery. The aim of this randomised controlled trial was to assess their contribution to the delivery of surgical care in rural hospitals in Zambia.

**Methods:**

Sixteen hospitals were randomly assigned to intervention and control arms of the study. Nine MLs were deployed to eight intervention sites. Crude numbers of selected major surgical procedures between intervention and control sites were compared before and after the intervention. Volume and outcomes of surgery were compared within intervention hospitals, between NPCs and surgically active medical doctors (MDs).

**Results:**

There was a significant increase in the numbers of caesarean sections (CS) in the intervention hospitals (+ 15.2%) and a drop by almost half in the control group (− 47%) (*P* = 0.015), between the two time periods. There were marginal shifts in the numbers of index procedures: a small drop in the intervention group (− 4.9%) and slight increase in the control arm (+ 4.8%) (*P* = 0.505). In all pairs, MLs had higher mean number of CS and other major surgical cases done in the intervention period compared with MDs. There was no significant difference in postoperative wound infection rates for CS (*P* = 0.884) and other major surgical cases (*P* = 0.33) at intervention hospitals between MLs and MDs.

**Conclusion:**

This study provided evidence that the ML training programme in Zambia is an effective and safe way to bridge the gap in rural hospitals between the demand and the limited availability of surgically trained workforce in the country. Such evidence is greatly needed as more developing countries are developing national surgical plans.

**Trial registration:**

ISRCTN66099597 Registered: 07/01/2014

## Introduction

The global shortage of surgeons disproportionately impacts low- and middle-income countries (LMICs) [1–3]. Zambia falls short of the 2006 World Health Report recommendation on the physician to population density of 22.8 per 10 000 [4, 5]. To mitigate chronic health workforce shortages, some countries train non-physician clinicians (NPCs) to perform functions normally carried out by doctors [6, 7], a response termed ‘task-shifting’ [8]. The concept has been recommended by the World Health Organization [9], but there are concerns about its safety for patients and ethical issues about patient’s rights to access the best possible care [10, 11]. Such concerns may be due to professional resistance and the possibility of competing interests between specialist and NPCs undertaking roles historically performed by the former [12]. Despite these concerns, countries such as Zambia have invested in training such cadres to fill the human resource gap in the delivery of surgical care [13]. NPCs can be trained to perform most surgical procedures at a district level, requiring shorter training times than doctors [14]. Studies have found that NPCs can carry out simple surgical procedures [7, 15–20]. In addition, studies comparing rates of postoperative complications between doctors and NPCs have found no significant differences [21–23]. NPCs also have a high retention rate in rural hospitals, reflecting their willingness (unlike doctors) to live in rural districts [8, 24].

Since 2002, NPCs, called medical licentiates (MLs) in Zambia, have been contributing to the delivery of essential surgical services at district level hospitals (DLHs), after completion of an advanced diploma course at the Chainama College of Health Sciences. The 2-year ML curriculum covered key topics in Biomedical Sciences, followed by hospital rotations for supervised training in Internal Medicine, Surgery, Paediatrics and Obstetrics and Gynaecology, and a 1-year internship. Over 200 MLs were trained, and in 2013, the programme was upgraded to the level of Bachelor of Science (4 years direct entry programme). However, to date, there has been no research on the effectiveness of using NPCs to widen availability of surgical services in rural areas.

The aim of this study was to assess the contribution of surgically trained MLs working in district hospitals. It was undertaken as part of the EU funded Clinical Officer Surgical Training in Africa (COST-Africa) project, implemented between 2011 and 2016 [13]. The project aimed to strengthen the surgical capacity of district hospitals by enhancing the surgical skills of MLs through a two-step intervention. The first step involved an intensive, 3-month course in surgery for MLs, funded by COST-Africa. The second component was a programme of quarterly supervision by specialist surgeons to oversee the COST-Africa-trained MLs, once they were deployed to intervention districts. Details of the intervention are reported elsewhere [13]. This paper was guided by the following question: how much surgery was undertaken by the COST-Africa-trained MLs at district hospitals?

## Methods

### Setting

A situation analysis was conducted in 2013 to assess DLHs’ capacity to deliver surgery, using a simplified version of the WHO tool for Situational Analysis to Assess Emergency and Essential Surgical Care [25]. This covered availability of essential surgical staff, infrastructure, equipment, drugs and consumables. All 84 district level hospitals (in Zambia called level 1 hospitals), countrywide, were contacted at least twice to take part in the survey. Fifty-four hospitals completed it. Hospitals were also asked to report numbers of caesarean sections (CS) and other major general surgical cases performed over the previous 12 months. The University of Zambia Biomedical Research Ethics Committee approved the project in 2011. Cluster consent was obtained from hospital managers to allow analysis of anonymised aggregated data and publication of findings.

Inclusion criteria comprised as follows: being a level 1 (public or mission) DLH with no practising ML, having a functioning operating theatre (surgical operations performed in the past 12 months) and self-reported potential to scale up surgery. Findings from the situation analysis (reported elsewhere [26]) were used to identify facilities suitable to participate in this study and to facilitate randomised allocation of DLHs to intervention and control groups. Following advice of the local research team, DLHs in the Lusaka and Copperbelt were excluded as not comparable with the ones in the other provinces. They were deemed to be better staffed with easier access to specialist facilities.

### Hospital selection and randomisation

Data from the situation analysis were entered into an MS Excel spreadsheet and binary scores applied according to the presence or absence of three sets of criteria: (i) evidence of essential equipment and a functioning operating theatre, (ii) presence of essential surgical and anaesthetic staff and (iii) total numbers of major and minor surgical cases in the previous 12 months. Specialist surgeons based at the nearest referral hospitals were asked to review the results of the situation analyses and confirm or discount the capacity of the DLHs to deliver major surgery, based on their direct knowledge of their district hospitals. Hospitals were scored as green (definitely eligible), orange (possibly eligible) or red (not eligible for deployment of a surgically active ML), through a consensus process involving researchers and surgically trained staff from the Zambian, Irish and Netherlands partners.

Eight DLHs hospital pairs were formed based on similarity in respect of four matching variables: number of caesarean sections, number of other major surgical cases, number of hospital beds and size of government annual grant. Pairs with the best match were established within provinces or within the referral network of a large tertiary hospital. The Ministry was then requested to create treasury positions (funded posts for MLs) so that COST-Africa graduates could be deployed to the designated intervention hospitals.

The study was designed as a matched-pairs randomised control trial (RCT) and registered retrospectively 07 Jan 2014, trial number: ISRCTN66099597. A simple randomisation method was used: the hospital names were written on 16 pieces of paper, which were paired; and a senior MoH representative selected one of each pair, blind to their names. All hospitals remained in the study for the duration of the trial.

The MLs were free to choose where to be deployed from the list of selected intervention district hospitals. Seven hospitals received one ML and one received two for logistical reasons. Due to a period of major reforms of government ministries, which delayed ML deployments, in some cases for many months, the project failed to ensure the same start date and equal periods of ML deployment in each pair. The duration of the intervention varied from 9 to 24 months, with a median of 17 months. Table 1 provides an overview of the intervention and control sites, and the duration of the intervention periods.

**Table 1:** COST-Africa pairs of selected hospitals (*MH* mission/faith-based hospital, *DH* government-owned district hospital), duration of the intervention period and number of trainees

Intervention	Control	Province	Intervention duration
St. Margaret MH	Lubwe MH	Luapula	22
Maamba DH*	Zimba MH	Southern	20
Mtendere MH	Mazabuka DH**	Southern	20
Choma DH**	Itezhi-Tezhi DH	Southern/Central	24
Mwinilunga DH	Chavuma MH	North Western	17
Serenje DH	Liteta DH	Central	12
Kalene MH	Kabompo DH	North Western	9
Siavonga DH	Sesheke DH	Southern/Western	8

*DLH which received two MLs

**Now (2019) general hospitals

### Primary outcome and randomisation

The primary outcome was the change in the number of common general surgical procedures performed at the participating hospitals before and after the deployment of MLs, with control DLHs (with no MLs) as a comparator. The index of common surgical procedures comprised: CS, hysterectomy, salpingectomy, laparotomy, hernia and hydrocele repair.

### Secondary outcomes

In the intervention hospitals, the study tracked surgical productivity (number of cases done) and quality of surgery (rates of surgical site infections [27]) of MLs, as secondary outcomes. Surgical productivity was measured as the percentage and mean number of procedures (CS and other index general surgical cases) performed by the MLs compared with medical doctors (MDs). MDs were the highest qualified cadres as no surgical specialists were stationed at these hospitals. Quality of surgery was evaluated in each hospital by comparing in-hospital perioperative mortality and wound infection rates of MLs and MDs, which is a common way used by others [28].

### Data collection

We collected data for 12 months prior to the deployment of the MLs and for the final 12 months of the intervention. In the two pairs where the intervention was shorter, the data collection period in the paired control hospital was also shorter (9 months for Kalene and 8 months for Siavonga—Table 1). The situation analysis had revealed a lack of routine monitoring of surgery at DLHs and an absence of standardised surgical data collection tools. The research team developed and piloted an extended theatre register (ETR) to collect data on surgical procedures and a tool to capture monthly summaries of procedures performed. The ETR, introduced in the intervention hospitals, was designed to capture basic data that should be collected routinely in operating theatres: patient demographics, indication for surgery, procedure, type of anaesthesia, surgical team composition and outcomes. It additionally collected variables such as postoperative complications (wound infection, urinary infection, respiratory infection), date of discharge, patient health status at discharge and final destination (home, referred to another hospital, or dead).

Control hospitals reported their surgical outputs using the monthly summary tool. At the end of the data collection period, surgical specialists were tasked to code the data submitted from intervention sites. To prevent misclassifications, lists of diagnoses and corresponding surgical procedures were agreed, with codes assigned to each diagnosis and procedure. Tools are available at http://costafrica.weebly.com/data-collection.html. At the end of the intervention, COST-Africa researchers visited all intervention and control hospitals, to verify the data and correct any errors.

### Statistical analysis

Relative percentage changes in the primary outcome before and after the intervention, and between the intervention and control hospitals, were examined using the Mann-Whitney *U* test. Chi-square tests were used to examine differences in surgical productivity between MLs, MDs and other surgically active cadres. Postoperative wound infection rates at intervention hospitals were compared between MLs and MDs using Fisher’s exact test. The analysis was undertaken separately for CS and the index of general surgical procedures commonly performed at district hospitals. All tests were two-tailed and statistical significance at *P* < 0.05 resulting with 95% confidence intervals.

## Results

### Primary outcome

There was a significant increase in the numbers of CS in the intervention hospitals and a drop by almost half in the control group, between the two time periods. There were marginal shifts in the numbers of index procedures: a small drop in the intervention group and slight increase in the control arm (Table 2).

**Table 2:** Change in crude numbers of CS and index procedures in intervention and control DLHs before and after the intervention

	Intervention (%change)		Control (%change)	
	Before	After	Before	After
CS	900	1037 (15.2%)	990	525 (− 47%)
Index	508	483 (− 4.9%)	417	437 (+ 4.8%)

Analysis of individual pairs of hospitals revealed no clear pattern for the primary outcome (Table 3). In five of eight pairs, the intervention hospitals outperformed the control ones in terms of the percentage change in numbers of CS undertaken (*P* = 0.015). For the index general surgical procedures, five control hospitals outperformed the intervention ones; however, the difference was not statistically significant (*P* = 0.505).

**Table 3:** Percentage change in CS and index procedures between 2013 and 2015 in intervention vs. control sites

Intervention	% change from 2013 to 2015 (n 2013 vs n 2015)		Control	% change from 2013 to 2015 (n 2013 vs n 2015)	
	CS	Index		CS	Index
Siavonga	− 12 (68 to 60)	55 (11 to 17)	Sesheke	− 31 (58 to 40)	− 65 (74 to 26)
Serenje	1184 (13 to 167)	300 (7 to 28)	Liteta	− 39 (87 to 53)	− 48 (23 to 12)
Kalene	29 (126 to 163)	− 16 (100 to 84)	Kabompo	− 67 (91 to 31)	− 15 (20 to 17)
Mwinilunga	60 (77 to 123)	0 (44 to 44)	Chavuma	− 34 (65 to 43)	12 (137 to 154)
Choma	− 34 (292 to 192)	− 21 (174 to 138)	Itezhi-Tezhi	2 (91 to 93)	22 (9 to 11)
Mtendere	− 6 (126 to 118)	− 1 (79 to 78)	Mazabuka	1 (172 to 174)	536 (11 to 70)
Maamba	4 (178 to 185)	− 6 (90 to 85)	Zimba	13 (197 to 223)	24 (91 to 113)
St. Margaret	45 (20 to 29)	200 (3 to 9)	Lubwe	− 59 (227 to 91)	− 35 (52 to 34)

### Productivity

A total of 6082 operations were done at the eight intervention hospitals in 2015. We compared the share performed by MDs with the share performed by MLs (Table 4). In all intervention hospitals, surgically active MLs were outnumbered by surgically active MDs. In four pairs, MLs did more index procedures than MDs, and in two pairs, MLs did more CSs than MDs.

**Table 4:** Percentage of 6082 operations in 2015 by cadre

Hospital	Cadre (number of staff)	Surgical procedures		
		CS	Index	Other***
Choma*	ML(1)	23.2%	26.1%	26.7%
	other(3)**	1.5%	4.9%	3.9%
	MD (12)	75.3%	69.0%	69.3%
Kalene	ML (1)	39.0%	60.8%	23.1%
	other(3)	0.5%	0.0%	15.8%
	MD(5)	60.5%	39.2%	61.1%
St. Margaret	ML (1)	65.2%	88.9%	63.0%
	other(3)	11.6%	0.0%	27.2%
	MD (2)	23.2%	11.1%	9.9%
Maamba	ML (2)	40.1%	37.1%	43.5%
	Other (3)	0.8%	0.7%	18.7%
	MD (7)	59.1%	62.1%	37.8%
Mtendere	ML (1)	51.5%	37.5%	56.8%
	MD (4)	48.5%	62.5%	43.2%
Mwinilunga	ML (1)	38.7%	29.2%	31.0%
	Other (3)	0.0%	1.5%	10.0%
	MD (3)	61.3%	69.2%	59.0%
Serenje	ML (1)	49.7%	69.2%	73.8%
	Other (2)	0.0%	0.0%	10.7%
	MD (2)	50.3%	30.8%	15.5%
Siavonga	ML (1)	41.1%	76.2%	48.2%
	MD(2)	58.9%	23.8%	51.8%
Total	ML	40.6%	39.7%	38.9%
	Other	1.0%	1.8%	10.9%
	MD	58.4%	58.5%	50.1%

*Difference not statistically significant (*P* = 0.155)

**Other cadres: surgically active clinical officers and theatre nurses

***Other procedures: foreign body removal, debridement/sloughectomy, circumcision, other major and minor obstetric procedures (unclassified), suturing, cataract removal, other major disability preventive procedures and other major injury-related procedures

Analysis of mean numbers of CS and index procedures done by the two cadres at individual hospital level demonstrated that the MLs compared to MDs performed more surgeries (Table 5).

**Table 5:** Mean number of operations per clinician in the intervention period at the intervention hospitals by cadre

Hospital	ML		MD	
	CS	Index	CS	Index
Siavonga	30	16	23	3
Serenje	86	18	44	4
Kalene	78	48	24	6
Mwinilunga	72	19	38	15
Choma	62	53	17	12
Mtendere	103	45	24	19
Maamba	76	26	32	12
St. Margaret	45	16	8	1

### Quality of surgery: outcomes and safety

There was no statistically significant difference in wound infection rates for operations performed by MLs and those performed by MDs, for both CS and index procedures (Table 6). There was one surgical death recorded in 2015, after an exploratory laparotomy done by a MD. Intra-hospital deaths prior to surgery, primarily associated with delayed major obstetrical emergencies, were not deemed surgical deaths [29].

**Table 6:** Wound infection rates for CS and index procedures compared between MLs and MDs in the intervention hospitals

	ML		MD	
	Yes	No	Yes	No
CS*	9 (1.7%)	535 (98.3%)	12 (1.6%)	758 (98.4%)
Index**	5 (2.8%)	176 (97.2%)	13 (5.6%)	219 (94.4%)

*P = 0.884, **P = 0.33

## Discussion

This study provides insights into the contribution of NPCs to providing essential surgical services in district and rural hospitals in Zambia, directly addressing a critical dimension of health equity [30]. It also provides evidence for policy makers that support the concept of training mid-level cadres to be surgical providers in the absence of MDs and surgical specialists. Findings of this RCT are complementary to our earlier qualitative study, which reported a range of positive effects of the MLs at these DLHs [13].

The main study objective was to demonstrate the contribution of MLs once deployed to district hospitals, in terms of surgical outputs, and secondly to establish if the surgical care delivered by MLs was safe in comparison with other surgically active cadres with higher qualifications. Such evidence is needed in countries unable to deploy and retain specialist surgeons at the district level, who require alternative safe surgical models to meet surgical need. Zambia, which has recently developed a National Surgical, Obstetric and Anaesthesia Plan (NSOAP) [31, 32], is well placed to benefit from such evidence.

The comparison of surgical output between intervention and control sites showed a slight increase in CSs performed in intervention facilities and a major drop in CSs in control facilities. Given the available data, we are unable to determine the reasons for these changes, including whether or not the deployment of surgically trained MLs had some protective effect in intervention hospitals, vis-a-vis control hospitals. We contacted hospital managers in the control arm of the study requesting them to explain the changes observed. Informal feedback suggests that the drop in CS rates was caused by a combination of staff turnover and general reduction in the number of deliveries in certain areas. CSs, in sub-Saharan countries such as Zambia, are in most cases performed in response to an obstetric emergency [33] and usually at the nearest available hospital with the capacity to undertake such operations [34]. Further research will be required to explain the trends in CSs.

The numbers of general index surgical procedures increased in only three intervention sites. This finding confirms results of our other study in Zambia, where increases in availability of surgical staff in DLHs had little or no effect on overall volume of surgical output [26]. In the current state of the Zambian health system, it may not be enough to focus only on producing and deploying more surgically capable health workers to DLHs, without also addressing other obstacles and gaps hindering service provision. In a follow-up study (publication forthcoming), we demonstrate that the greatest shortage at the district level in Zambia is in the number anaesthesia providers. This particular shortage is a leading cause of unnecessary referrals of general surgical cases. Other obstacles include surgical infrastructure and medical supplies [26].

These findings are relevant for future policy making, because one of the main objectives of the Zambian NSOAP 2017-2021 is to increase the numbers of surgical providers [35]; and anaesthesia providers may need to be prioritised. An alternative explanation for reductions in surgical outputs in the control hospitals is that that there was little or no unmet need for surgery, or there was a considerable need but people were not presenting to these hospitals. Some support for the under-utilisation of available surgical services, as an explanation, comes from MoH reports, reporting a relative high number of hospitals that can provide surgical services [36] in what is a low-density population [37]. If future population studies demonstrate an unmet need for surgery in rural communities, there might be a need to stimulate demand through sensitisation campaigns informing rural dwellers about availability of surgical services in DLHs. It is also possible that other hospitals outside the study sample absorbed some of the demand; however, in our situation analysis, we did not observe any high-performing hospitals which performed considerably more surgeries than the ones in the study sample.

In a similar study conducted in Malawi, we found that the NPCs in the intervention arm increased surgical productivity by 74% after deployment [23]. Similar results were also observed in a study in Sierra Leone [28]. Both interventions, in Malawi and Sierra Leone, appeared to be responding to an unmet need. Further research is needed to establish the actual need for surgery in Zambia and patient flow patterns. When such data are available, there may be a case for prioritising scale up of surgical services at the best-performing facilities, where patients are frequent and where case volume is sufficient to maintain quality. However, equity and access for more remote rural populations must still be monitored.

This study investigated task-shifting and found that at an individual hospital level, MLs performed more surgery than MDs and other cadres, confirming studies from Malawi and Mozambique [23, 38]. It was clear from a qualitative substudy that the deployment of surgically trained MLs freed up time for MDs to do other clinical work [13]. Perhaps the intervention had spillover effects impacting on indicators which had not been originally planned and measured in the study design. A follow-up study [39] may provide more insight into the team dynamics between MLs and MDs to quantify the range and scale of benefits and challenges of ‘task-shifting’ major surgical services in these settings.

NPCs such as MLs are often considered a substitute for medically qualified professionals, and concerns around the safety of surgical ‘task-shifting’ solutions have been expressed [40]. Our findings revealed no difference in patient outcomes, between MLs and MDs, in the same hospital setting, using wound infections and surgical mortality as measures. This provides some evidence that ‘task-shifting’ is a safe way to bridge the gap in rural hospitals between the demand and the limited availability of surgically trained workforce.

### Limitations

The study had limitations. Firstly, there could have been a selection bias, because 30 hospitals out of the 84 sampled for the situation analysis, in 2011–2012, did not provide information on their capacity to deliver surgery. Secondly, an initial agreement with the Ministry of Health was undermined when responsibility for deployment of staff to DLHs was transferred to a different government ministry in 2012. This led to major delays in deploying surgically trained MLs to the intervention hospitals and different timelines and durations for the intervention. MLs with shorter stays at intervention hospitals had less opportunity to achieve significant increases in surgical outputs. Responsibility for staff deployment was returned to the MoH in August 2015, 5 months before the end of the intervention. This experience illustrates the difficulties in conducting health systems’ intervention research, especially RCTs, in real-life settings, involving a government salaried cadre at the forefront of routine health care delivery. Secondly, data collection in the intervention sites was done by the MLs participating in the study. To minimise the chance of bias, the project researchers verified the data at the end of the project through a series of visits to all hospitals. Nevertheless, the study findings demonstrate the feasibility of a model of surgical care at district hospitals using trained and supervised NPCs, with the potential to provide a sustainable safe surgical service to rural populations.

## Conclusions

Our research provides empirical evidence that the national training model for NPCs, launched in Zambia almost two decades ago, is effective and is achieving its aims. Such evidence is greatly needed, because there is no universal consensus around ‘task-shifting’ in surgical care [10, 41], and very little published in respect to outputs and outcomes. Our evidence supports the priorities of the Zambian NSOAP, which recognises the importance of training MLs, along with the training of other NPCs such as anaesthesia clinical officers in particular [35]. Further research is needed to establish the long-term impact of the training model on population health in Zambia and to determine the effects of upgrading the programme to a BSc level on ML career choices and retention of this cadre in rural areas of the country.

## Data Availability

The datasets generated and/or analysed during the current study are not publicly available due to its confidential nature and potential sensitivity issues resulting from data disclosure, but are available from the corresponding author on reasonable request.

## Abbreviations

BSc: Bachelor of Science
COST-Africa: Clinical Officer Surgical Training in Africa
CS: Caesarean section
DLH: District level hospital
ETR: Extended theatre register
LMIC: Low- and middle-income country
MD: Medical doctor
ML: Medical licentiate
MoH: Ministry of Health
NPC: Non-physician clinician
NSOAP: National Surgical, Obstetric and Anaesthesia Plan
RCT: Randomised control trial
